# Nomogram to predict postoperative infectious complications after surgery for colorectal cancer: a retrospective cohort study in China

**DOI:** 10.1186/s12957-021-02323-1

**Published:** 2021-07-08

**Authors:** Jing Wen, Tao Pan, Yun-chuan Yuan, Qiu-shi Huang, Jian Shen

**Affiliations:** 1 Department of Gastrointestinal Surgery, Chengdu Second People’s Hospital, No. 10 Qinyun Nan Street, Chengdu, 610041 Sichuan Province China; 2 Department of Gastrointestinal Surgery, Sichuan Cancer Hospital & Research Institute, School of Medicine, University of Electronic Science and Technology of China (UESTC), Chengdu, China; 3 College of Basic Medicine, Chongqing Three Gorges Medical College, Chongqing, China

**Keywords:** Colorectal cancer, Infectious complications, Risk mode

## Abstract

**Background:**

Postoperative infectious complications (ICs) after surgery for colorectal cancer (CRC) increase in-hospital deaths and decrease long-term survival. However, the methodology for IC preoperative and intraoperative risk assessment has not yet been established. We aimed to construct a risk model for IC after surgery for CRC.

**Methods:**

Between January 2016 and June 2020, a total of 593 patients who underwent curative surgery for CRC in Chengdu Second People’s Hospital were enrolled. Preoperative and intraoperative factors were obtained retrospectively. The least absolute shrinkage and selection operator (LASSO) method was used to screen out risk factors for IC. Then, based on the results of LASSO regression analysis, multivariable logistic regression analysis was performed to establish the prediction model. Bootstraps with 300 resamples were performed for internal validation. The performance of the model was evaluated with its calibration and discrimination. The clinical usefulness was assessed by decision curve analysis (DCA).

**Results:**

A total of 95 (16.0%) patients developed ICs after surgery for CRC. Chronic pulmonary diseases, diabetes mellitus, preoperative and/or intraoperative blood transfusion, and longer operation time were independent risk factors for IC. A prediction model was constructed based on these factors. The concordance index (C-index) of the model was 0.761. The calibration curve of the model suggested great agreement. DCA showed that the model was clinically useful.

**Conclusion:**

Several risk factors for IC after surgery for CRC were identified. A prediction model generated by these risk factors may help in identifying patients who may benefit from perioperative optimization.

## Introduction

Colorectal cancer (CRC) is among the commonest malignancies worldwide [1–3]. Surgical resection is considered the best choice for a potentially radical cure [4–7]. Even with advances in surgical techniques and perioperative treatment in recent years, mortality and morbidity rates after CRC surgery remain considerable, mainly due to postoperative infectious complications (ICs) [8, 9]. ICs after surgery for CRC have been demonstrated to increase cost, hospital stays, and delay the initiation of adjuvant treatments [10]. Importantly, multiple studies have shown that they are associated with decreased long-term survival [10–13].

Possible explanations for the relationship between postoperative ICs and oncological outcome include (1) escape of intraluminal neoplastic cells in patients with anastomotic leak [11], (2) local and systemic proliferation of proinflammatory cytokines and mediators [11, 14], (3) the association of ICs with increased TNM stage [15], (4) delays in the initiation of adjuvant treatments [11], and (5) poor surgical technique, which may increase the incidence of ICs and tumor recurrence. A better understanding of risk factors associated with ICs after surgery for CRC can aid healthcare providers in preoperative counseling and surgical decision-making, suggest complication-reducing strategies, and help in considering preventative measures.

Therefore, we designed the study to identify risk factors for ICs after surgery for CRC. We also used the risk factors to generate a nomogram that can predict the probability of postoperative ICs. We chose to evaluate preoperative and intraoperative factors because this model would be more clinically friendly and useful than models based on postoperative factors when ICs would be imminent. To our knowledge, this is the first prediction model that could predict the possibility of postoperative IC after surgery for CRC.

## Method

### Study population and ethical issues

Between January 2016 and April 2020, 593 consecutive patients who underwent surgery for primary CRC at Chengdu Second People’s Hospital were enrolled in the study. The inclusion criteria were (1) histologically confirmed CRC, (2) patients underwent surgery for CRC with radical resection, (3) patients had resection with a primary anastomosis without a protecting stoma, and (4) patients over 18 years old. The exclusion criteria were (1) palliative surgery, (2) with local surgical treatment (such as trans-anal endoscopic microsurgery), (3) with a stoma (such as Hartmann’s procedure, abdominoperineal resection, and anastomosis with a de-functioning stoma), (4) patients less than 18 years old, (5) with emergency surgery, (6) with evidence of infection or systemic autoimmune disease before surgery, and (7) with incomplete medical data. Patient data were extracted from a prospectively maintained CRC database. This study was approved by the Ethics Commission of the hospital (Chengdu Second People’s Hospital).

### Clinicopathological materials

Various preoperative and intraoperative variables were collected for risk factor selection as follows: basic information: sex, age, body mass index, smoking history, the American Society of Anesthesiologists (ASA) score, pre-existing co-morbidities (including heart disease, hypertension requiring medication, chronic pulmonary disease, diabetes mellitus), previous abdominal surgery, neoadjuvant chemo-radiotherapy, and preoperative and/or intraoperative blood transfusion; laboratory tests information: preoperative hemoglobin and albumin level; tumor information: preoperative TNM stage, tumor location, and tumor size; and surgical information: surgical approach, combined organ resection, intraoperative blood loss, and operation time.

Preoperative staging evaluation included digital rectal examination, rectal endosonography, colonoscopy, and MRI or CT scans. The indication for blood transfusion was a hemoglobin level below 80 g/L. When the hemoglobin level was between 80 and 100 g/L, blood transfusion was selected based on hemodynamics and oxygen saturation [16]. The operations in the study were performed by two surgeons (S.J., and B.J.). Both of them are attending doctors and have at least 14 years of experience in gastrointestinal surgery. Each of them performs at least 230 operations for gastric and colorectal cancer annually since 2015.

### Definition of postoperative infectious complications

In the present study, ICs were graded according to the Clavien-Dindo surgical complication system [17]. When a patient had at least two ICs, the higher grade was adopted [18]. ICs were defined as Clavien-Dindo grade II or more severe. ICs included wound infection, anastomosis leakage, intra-abdominal abscesses and collections, cholecystitis, infectious diarrhea, and pneumonia.

(1) Wound infection was confirmed when it gets painful with pustular discharge and/or a positive culture, the opening of the wound, and antibiotic treatment was required. (2) Anastomotic leakage was considered if any of the following situations were observed: fecal or gas discharge from the drain tract, vagina, or the incisional wound; fecal peritonitis; or peritonitis along with anastomotic defect confirmed by rectal examination, endoscopy, laparotomy, or radiological findings [19]. (3) Intra-abdominal abscesses and collections were confirmed by ultrasonography or computed tomography (CT) scans, accompanied by systemic inflammatory response lasting for at least 24 h [20]. (4) Infectious diarrhea was diagnosed when a stool culture was positive for microbial pathogens and antibiotic treatment was required. (5) Cholecystitis was confirmed by CT scans or ultrasonography and accompanied by clinical signs and symptoms. (6) Pneumonia was defined as fever above 38.5 °C and positive radiological findings, requiring antibiotic treatment.

### Statistical analysis

Statistical analysis was performed using SPSS 19.0 (SPSS®, Chicago, IL, USA) and R software (Version 3.6.1; https://www.r-project.org). Categorical variables are represented by number and percentage, and continuous variables are represented by mean ± standard deviation. Categorical data were compared with Fisher’s exact test or Pearson χ^2^ test, and continuous data were compared with Mann–Whitney U test or independent sample *t*-test as appropriate.

We used the least absolute shrinkage and selection operator (LASSO) method to find the optimal variables with non-zero coefficients as risk factors [21]. Then, based on the results of LASSO regression analysis, multivariable logistic regression analysis was used to establish a prediction model, and a nomogram was generated. Bootstraps with 300 resamples were performed for internal validation. The predictive performance was assessed by Harrell’s concordance index (C-index). A calibration curve was plotted to evaluate the calibration of the nomogram. A decision curve analysis (DCA) was created to evaluate the clinical usefulness of the nomogram. *P*-value of < 0.05 was considered significant.

## Results

During the study period, a total of 593 patients who underwent colorectal surgery met the inclusion criteria. Among them, 95 patients (16.0%) developed postoperative ICs, including 1.0%, 2.9%, 3.5%, 0.2%, 1.3%, and 8.9% in wound infection, anastomotic leakage, intra-abdominal abscesses and collections, cholecystitis, infectious diarrhea, and pneumonia, respectively. Using univariate analysis, an older age (*p* = 0.035), ASA score 3 or 4 (*p* = 0.011), chronic pulmonary disease (*p* < 0.001), diabetes mellitus (*p* < 0.001), a lower preoperative hemoglobin level (*p* < 0.001), tumor location (*p* < 0.001), preoperative and/or intraoperative blood transfusion (*p* < 0.001), more intraoperative blood loss (*p* = 0.014), and a longer operation time (*p* < 0.001) were identified as significant risk factors for IC (Table 1). The detailed information of ICs is shown in Table 2.

**Table 1:** The baseline characteristics of the patients with and without ICs

Variables		IC		p†
		With (*n* = 95)	Without (*n* = 498)	
Sex	Male	58 (9.8%)	279 (47.0%)	0.365
	Female	37 (6.2%)	219 (37.0)	
Age*	Year	69.2±10.1	66.6±11.2	0.035‡
BMI*	kg/m^2^	22.9±3.4	22.7±3.3	0.557‡
Smoking history	Yes	18 (3.0%)	97 (16.4%)	0.905
	No	77 (13.0%)	401 (67.6%)	
ASA score	< 3	79 (13.3%)	456 (76.9%)	0.011
	≥3	16 (2.7%)	42 (7.1%)	
Heart disease	Yes	5 (0.8%)	34 (5.7%)	0.573
	No	90 (15.2%)	464 (78.3%)	
Hypertension	Yes	27 (4.6%)	161 (27.2%)	0.453
	No	68 (11.5%)	337 (56.8%)	
Chronic pulmonary disease	Yes	28 (4.7%)	30 (5.1%)	< 0.001
	No	67 (11.3%)	468 (78.9%)	
Diabetes mellitus	Yes	30 (5.1%)	59 (9.9%)	< 0.001
	No	65 (11.0%)	439 (74.0%)	
Previous abdominal surgery	Yes	30 (5.1%)	143 (24.1%)	0.574
	No	65(11.0%)	355 (59.9%)	
Neoadjuvant chemo-radiotherapy	Yes	12 (2.0%)	51 (8.6%)	0.488
	No	83 (14.0%)	447 (75.4%)	
Preoperative hemoglobin	g/l	104.5±30.1	118.5±24.4	<0.001‡
Preoperative serum albumin	g/l	43.5±13.2	41.7±16.7	0.475‡
Preoperative T stage	< 3	11 (1.9%)	76 (12.8%)	0.353
	≥3	84 (14.1%)	422 (71.2%)	
Preoperative N stage	Negative	57 (9.6%)	295 (49.7%)	0.890
	Positive	38 (6.4%)	203 (34.2%)	
Preoperative TNM stage	I	11 (1.9%)	69 (11.6%)	0.788
	II	45 (9.9%)	221 (37.3%)	
	III	39 (6.6%)	208 (35.1%)	
Tumor Size*	cm	4.7±1.4	4.5±1.5	0.357‡
Tumor Location	Right colon	20 (3.4%)	110 (18.5%)	<0.001
	Transverse colon	9 (1.5%)	7 (1.2%)	
	Left colon	9 (1.5%)	35 (5.9%)	
	Sigmoid	14 (2.4%)	87 (14.7%)	
	Rectum	43 (7.3%)	259 (43.7%)	
Blood transfusion^★^	Yes	29 (5.0%)	47 (7.9%)	<0.001
	No	66 (11.1%)	451 (76.1%)	
Surgical approach	Laparoscopic	33 (5.6%)	206 (34.7%)	0.227
	Open	62 (10.5%)	292 (4.2%)	
Combined organ resection	Yes	2 (0.3%)	18 (3.0%)	0.455
	No	93 (15.7%)	480 (80.9%)	
Intraoperative blood loss	ml	168±61.6	118.2±55.4	0.014‡
Operation time	min	229.6±68.8	204.1±56.6	<0.001‡
Number of retrieved lymph nodes	-	15.4±2.9	15.1±3.4	0.412‡

Values in parentheses are percentages unless indicated otherwise

*IC* infectious complication, *BMI* body mass index, *ASA* American Society of Anesthesiologists

*values are mean ± standard deviation

^★^Preoperative and/or intraoperative blood transfusion

†χ^2^ test

‡Paired t test

**Table 2:** Detailed information of postoperative ICs in the total population

IC	N (%)
Total^★^	95 (16.0%)
Wound infection	6 (1.0%)
Anastomosis leakage	17 (2.9%)
Intra-abdominal abscesses and collections	21 (3.5%)
Cholecystitis	1 (0.2%)
Infectious diarrhea	8 (1.3%)
Pneumonia	53 (8.9%)
Clavien-Dindo classification	
II	64 (10.8%)
III	16 (2.7%)
IV	13 (2.2%)
V*	2 (0.3%)

Values in parentheses are percentages; *IC* infectious complication

^★^Six patients had both anastomosis leakage and pneumonia; four patients had both intra-abdominal abscesses and pneumonia; one patient had both infectious diarrhea and pneumonia

*One patient died of sepsis caused by anastomotic leakage and one patient died of respiratory failure caused by severe pneumonia

### Risk factor selection

**Fig. 1:**
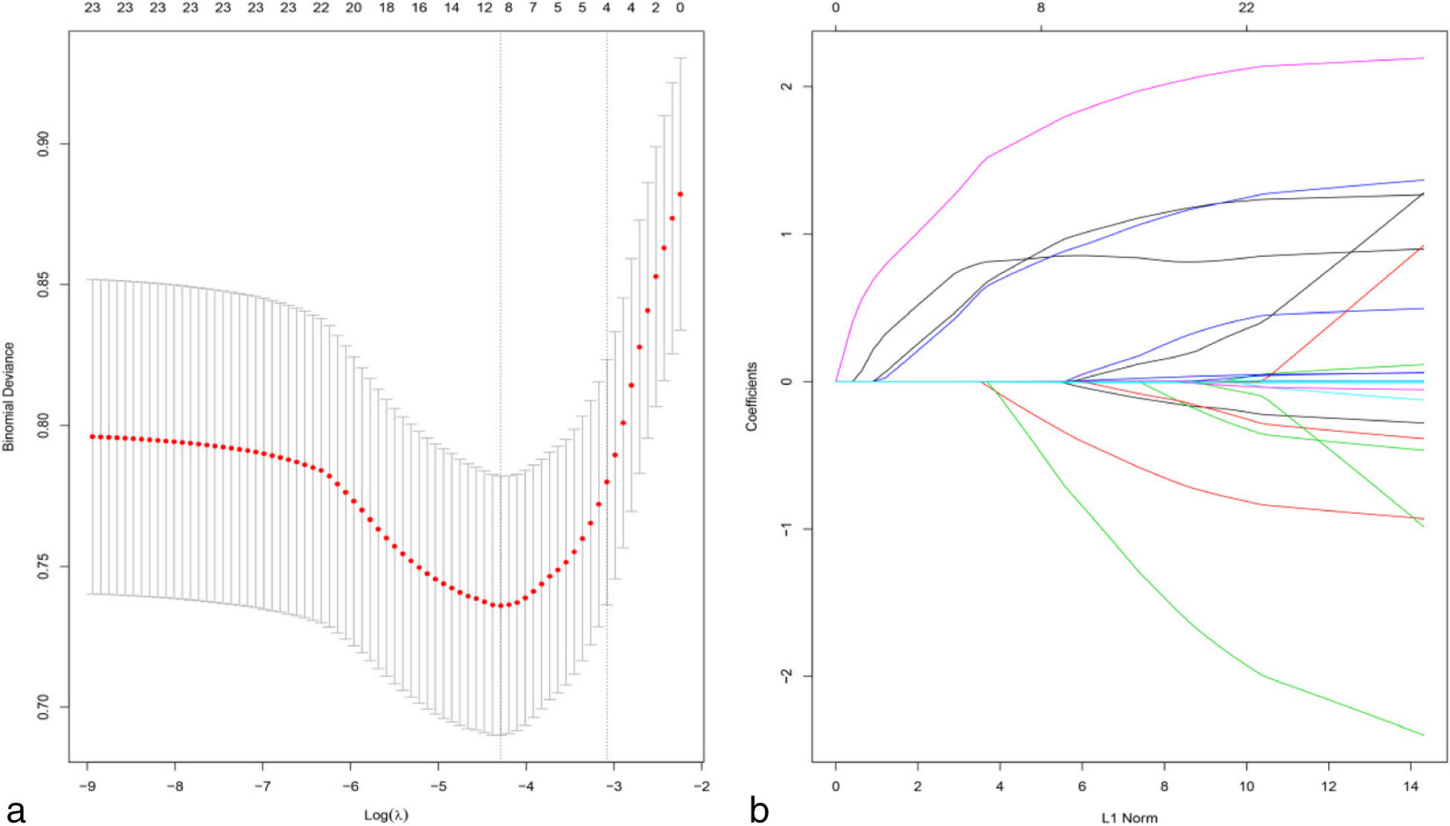
Risk factors selection using the least absolute shrinkage and selection operator (LASSO) logistic regression model. Final risk factors include chronic pulmonary disease, diabetes mellitus, preoperative and/or intraoperative blood transfusion, and longer operation time. **a** Optimal parameter (λ) selection in the LASSO model used five-fold cross-validation and minimum criteria. The partial likelihood deviance (binomial deviance) curve was plotted versus log(λ). Dotted vertical lines were drawn at the optimal values by using the minimum criteria and the 1 SE of the minimum criteria (the 1-SE criteria). **b** LASSO coefficient profiles of the 26 features. A coefficient profile plot was plotted against the log (λ) sequence, and the 4 non-zero coefficients were chosen at the values selected using fivefold cross-validation. SE, standard error

We used the LASSO regression analysis to evaluate the 24 variables (Fig. 1). Finally, we screened out 4 variables with nonzero coefficients as potential risk factors of postoperative ICs. These risk factors included chronic pulmonary disease, diabetes mellitus, preoperative and/or intraoperative blood transfusion, and longer operation time.

### Nomogram and validation

**Fig. 2:**
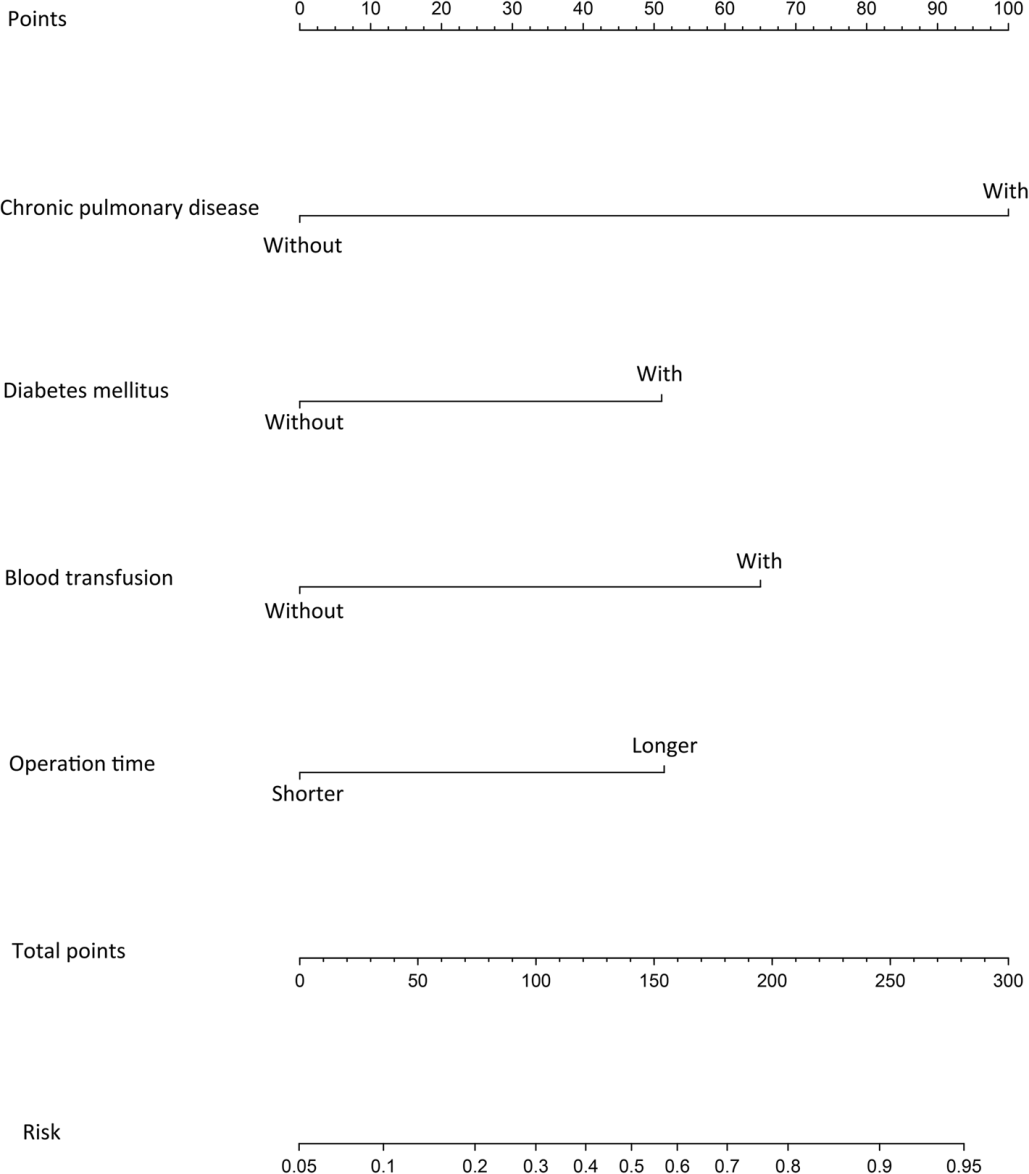
Nomogram for predicting IC following surgery for CRC. The nomogram was generated based on chronic pulmonary disease, diabetes mellitus, preoperative and/or intraoperative blood transfusion, and longer operation time

**Fig. 3:**
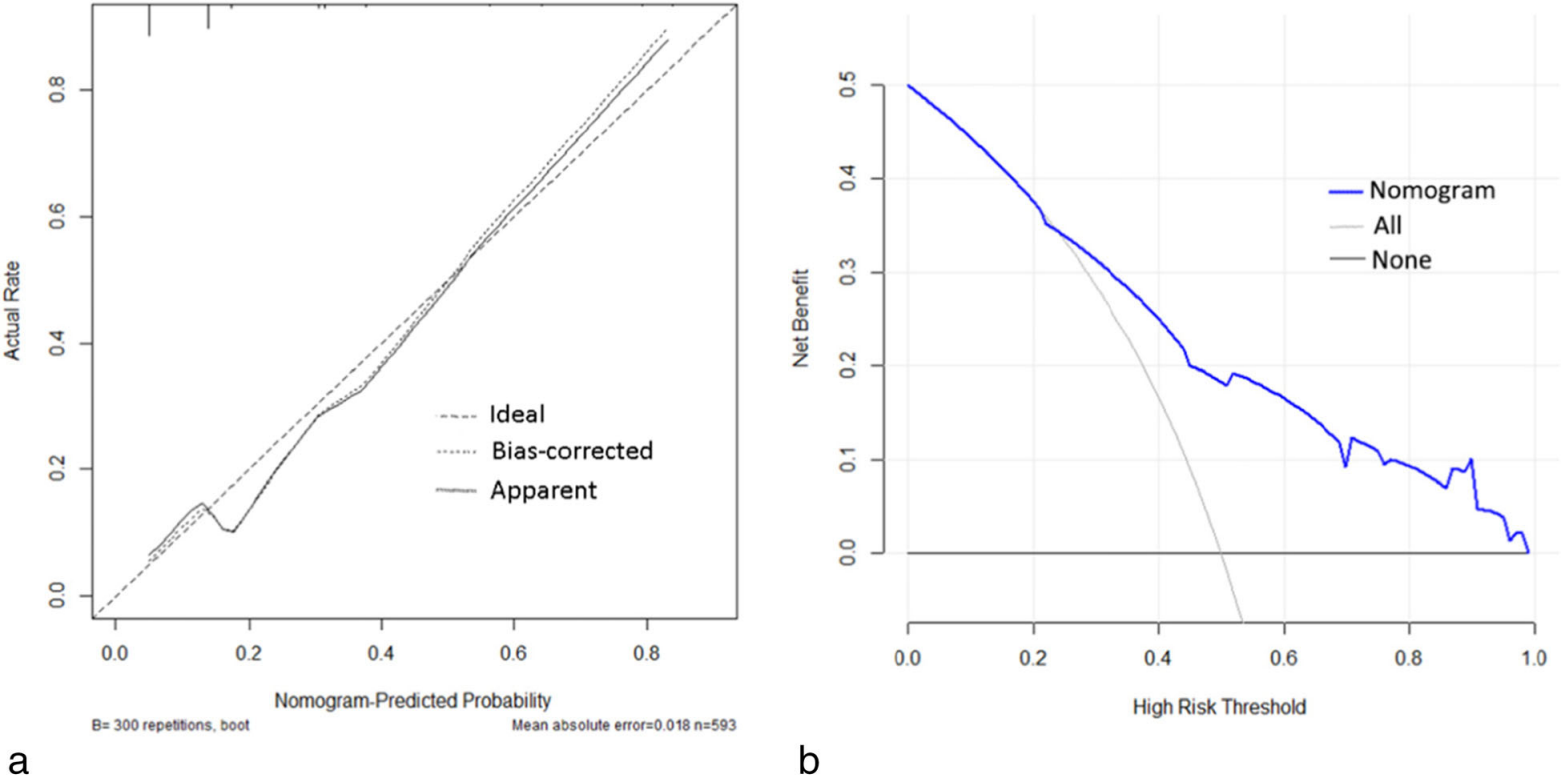
**a** Calibration curve of the nomogram for predicting IC following surgery for CRC. **b** Decision curve analysis for predicting IC following surgery for CRC. The x-axis shows the threshold probability. The y-axis represents net benefit. “None” to the assumption that no patient developed IC and “All” refers to the assumption that all patients developed IC. When the score is greater than 0.23, using the nomogram to predict IC adds more net benefit than the treat-none or treat-all strategies

We further conducted a multivariable logistic regression analysis and generated a prediction model to get a deep insight into the relationship between ICs and these risk factors. The results of the multiple logistic regression analysis are shown in Table 3 and visualized in the form of a nomogram to guide healthcare providers in the clinic (Fig. 2). The results showed that chronic pulmonary disease [hazard ratio (HR)=8.10, 95% confidence interval (95%CI): 4.22–15.56, *p*< 0.001], diabetes mellitus (HR=2.91, 95%CI: 1.61–5.26, *p*< 0.001), preoperative and/or intraoperative blood transfusion (HR=2.93, 95%CI: 1.78–4.84, *p*< 0.001), and longer operation time (HR=3.90, 95%CI: 2.13–7.12, *p*< 0.001) were independent risk factors for IC. The C-index of the nomogram was 0.761. The calibration curve of the nomogram suggested great agreement (Fig. 3). To use the nomogram, first, draw a vertical line to the top points row to assign points for each factor, and then, add the points from each factor together and drop a vertical line from the total points row to get the risk of IC.

**Table 3:** Risk factors for IC following surgery for CRC

Risk factors	β-coefficient	HR (95% CI)	P
Chronic pulmonary disease (with vs without)	2.09	8.10 (4.22–15.56)	<0.001
Diabetes mellitus (with vs without)	1.07	2.91 (1.61–5.26)	<0.001
Blood transfusion^★^ (with vs without)	1.08	2.93 (1.78–4.84)	<0.001
Operation time (longer vs shorter)	1.36	3.90 (2.13–7.12)	<0.001

*IC* infectious complication, *CRC* colorectal cancer, *HR* hazard ratio, *CI* confidence interval

^★^Preoperative and/or intraoperative blood transfusion

### Clinical usefulness

The decision curve analysis for the nomogram is shown in Fig. 3B. It showed that using the nomogram to predict ICs following surgery for CRC added more net benefit than the treat-all or treat-none strategies when the threshold probability is greater than 0.23.

## Discussion

IC remains the most significant cause of early morbidity and it decreases long-term survival after surgery for CRC [10, 22, 23]. Therefore, early recognition and prevention of IC in high-risk patients is an important issue. In the present study, a considerable number of patients (16.0%) developed ICs after surgery for CRC, which is comparable to multiple previous studies [24–27]. Furthermore, chronic pulmonary diseases, diabetes mellitus, preoperative and/or intraoperative blood transfusion, and longer operation time were identified as independent risk factors for ICs. A satisfactory model for ICs was also constructed based on these risk factors. The model can be used to target IC prevention and monitor interventions beyond standard infection prevention in high-risk patients who are likely to benefit.

In the present study, patient-related factors (chronic pulmonary disease and diabetes mellitus) were identified as independent risk factors for IC after surgery for CRC, which is in well agreement with previous literature [28–30]. Therefore, special attention should be paid to patients with these co-morbidities and we believe that preoperative treatment of these co-morbidities is essential for postoperative recovery in CRC patients.

As an indicator of the complexity and difficulty of the operation [31], our data validate previous studies that longer operation time is an independent predictor for IC [25, 32, 33]. Longer operation time may increase susceptibility to infection, resulting in IC development after surgery for CRC [7, 25]. Blood transfusion was another independent risk factor for IC. These findings were consistent with a previous study [34]. Although blood transfusion can improve oxygen delivery capacity and tissue perfusion in patients with severe anemia, it may also lead to systemic inflammation and other transfusion-related adverse events, particularly acute lung injury and infection [35, 36]. Furthermore, preoperative and intraoperative blood transfusions may reflect the patient's poor systemic condition or complexity of the surgery [37]. Therefore, special attention should be paid to CRC patients who have a blood transfusion in the perioperative period.

In the present study, we constructed a model to predict the possibility of IC after surgery for CRC. Healthcare providers could make individualized predictions of the IC probability with this model, which aligns with the current concept of personalized medicine [38]. Knowledge of the risk factors for IC would allow intervening in two ways: prevention and rigorous follow-up in high-risk patients after surgery. Prevention can be achieved by preoperative optimization of some high-risk conditions and correcting risk factors such as chronic pulmonary disease using bronco-dilatator treatment before surgery. A rigorous postoperative follow-up could allow the early recognition of IC, thus enabling its early intervention.

The strengths of the study are that it included a wide range of potential risk factors for IC. The proposed model was created based on routinely collected perioperative information to maximize its application and generalizability. Furthermore, we used the LASSO regression to identify risk factors for IC. LASSO regression allows selecting factors to include in the regression model, avoiding the usual methods of automatic factor selection (such as forward, backward and stepwise method), which have been previously reported to give wrong results in some situations [21]. Our study also had some limitations. First, the retrospective nature of the study may introduce bias. Prospective studies are needed to validate the prediction model. Second, the study was only a single-center study and the results were internally validated, external validation is needed to determine whether the results can be applied to other institutions.

## Conclusion

Several risk factors for IC after surgery for CRC were identified. A prediction model generated by these risk factors may help in identifying patients who may benefit from perioperative optimization.

## Data Availability

The datasets used and/or analyzed during the current study are available from the corresponding author on reasonable request.

## Abbreviations

IC: Infectious complication
CRC: Colorectal cancer
LASSO: Least absolute shrinkage and selection operator
DCA: Decision curve analysis
C-index: Concordance index
HR: Hazard ratio
CI: Confidence interval

## References

[1] Arnold M, Sierra MS, Laversanne M, Soerjomataram I, Jemal A, Bray F (2017). Global patterns and trends in colorectal cancer incidence and mortality. Gut..

[2] Liao CK, Yu YL, Lin YC (2021). Prognostic value of the C-reactive protein to albumin ratio in colorectal cancer: an updated systematic review and meta-analysis. World J Surg Oncol..

[3] Hou S, Wang Q, Zhao S, Liu F, Guo P, Ye Y (2021). Safety and efficacy of side-to-end anastomosis versus colonic J-pouch anastomosis in sphincter-preserving resections: an updated meta-analysis of randomized controlled trials. World J Surg Oncol.

[4] Hashiguchi Y, Muro K, Saito Y (2020). Japanese Society for Cancer of the Colon and Rectum (JSCCR) guidelines 2019 for the treatment of colorectal cancer. Int J Clin Oncol.

[5] Teurneau-Hermansson K, Svensson Neufert R, Buchwald P, Jorgren F (2021). Rectal washout does not increase the complication risk after anterior resection for rectal cancer. World J Surg Oncol.

[6] Papila Kundaktepe B, Papila C (2021). The clinical significance of preoperative plasma fibrinogen levels and platelet counts in resectable colon cancer. World J Surg Oncol.

[7] Kazama K, Numata M, Aoyama T (2021). Does the Endoscopic Surgical Skill Qualification System improve patients’ outcome following laparoscopic surgery for colon cancer? A multicentre, retrospective analysis with propensity score matching. World J Surg Oncol.

[8] Rovera F, Dionigi G, Boni L (2007). Infectious complications in colorectal surgery. Surg Oncol..

[9] Alsaif SH, Rogers AC, Pua P (2021). Preoperative C-reactive protein and other inflammatory markers as predictors of postoperative complications in patients with colorectal neoplasia. World J Surg Onco.

[10] Artinyan A, Orcutt ST, Anaya DA, Richardson P, Chen GJ, Berger DH (2015). Infectious postoperative complications decrease long-term survival in patients undergoing curative surgery for colorectal cancer: a study of 12,075 patients. Ann Surg.

[11] Eberhardt JM, Kiran RP, Lavery IC (2009). The impact of anastomotic leak and intra-abdominal abscess on cancer-related outcomes after resection for colorectal cancer: a case control study. Dis Colon Rectum..

[12] Watt DG, McSorley ST, Park JH, Horgan PG, McMillan DC (2017). A Postoperative Systemic Inflammation Score Predicts Short- and Long-Term Outcomes in Patients Undergoing Surgery for Colorectal Cancer. Ann Surg Oncol..

[13] Ide S, Okugawa Y, Omura Y (2021). Geriatric nutritional risk index predicts cancer prognosis in patients with local advanced rectal cancer undergoing chemoradiotherapy followed by curative surgery. World J Surg Oncol.

[14] Doherty J, Mcgregor J, Purdie C (1995). Efficacy of tumoricidal agents in vitro and in vivo. Br J Surg.

[15] Jérôme B, Guillaume P, William B (2013). Advanced tumor stage is an independent risk factor of postoperative infectious complications after colorectal surgery: arguments from a case-matched series. Dis Colon Rectum..

[16] Practice Guidelines for Perioperative Blood Transfusion and Adjuvant Therapies. Anesthesiology. 2006;105:198–208. 10.1097/00000542-200607000-00030 16810012

[17] Dindo D, Demartines N, Clavien PA (2004). Classification of surgical complications: a new proposal with evaluation in a cohort of 6336 patients and results of a survey. Ann Surg.

[18] Kubota T, Hiki N, Sano T (2014). Prognostic significance of complications after curative surgery for gastric cancer. Ann Surg Oncol..

[19] Yeh CY, Changchien CR, Wang JY (2005). Pelvic drainage and other risk factors for leakage after elective anterior resection in rectal cancer patients: a prospective study of 978 patients. Ann Surg.

[20] Moyes LH, Leitch EF, McKee RF, Anderson JH, Horgan PG, McMillan DC (2009). Preoperative systemic inflammation predicts postoperative infectious complications in patients undergoing curative resection for colorectal cancer. Br J Cancer..

[21] Jerome F, Trevor H, Rob T (2010). Regularization paths for generalized linear models via coordinate descent. J Stat Softw..

[22] Artiles-Armas M, Roque-Castellano C, Farina-Castro R, Conde-Martel A, Acosta-Merida MA, Marchena-Gomez J (2021). Impact of frailty on 5-year survival in patients older than 70 years undergoing colorectal surgery for cancer. World J Surg Oncol.

[23] Koo CY, Tai BC, Chan DKH, Tan LL, Tan KK, Lee CH (2021). Chemotherapy and adverse cardiovascular events in colorectal cancer patients undergoing surgical resection. World J Surg Oncol.

[24] Tatsuoka T, Okuyama T, Takeshita E, et al. Early detection of infectious complications using Creactive protein and the procalcitonin levels after laparoscopic colorectal resection: a prospective cohort study. Surg Today. 2021;51(3). 10.1007/s00595-020-02111-6 PMC7892676 32785845

[25] Kamonvarapitak T, Matsuda A, Matsumoto S (2020). Preoperative lymphocyte-to-monocyte ratio predicts postoperative infectious complications after laparoscopic colorectal cancer surgery. Int J Clin Oncol.

[26] van den Berg I, van den Braak RRJC, van Vugt JLA, Ijzermans JNM, Buettner S (2021). Actual survival after resection of primary colorectal cancer: results from a prospective multicenter study. World J Surg Oncol.

[27] Tang B, Lei X, Ai J, Huang Z, Shi J, Li T (2021). Comparison of robotic and laparoscopic rectal cancer surgery: a meta-analysis of randomized controlled trials. World J Surg Oncol.

[28] Masoomi H, Kang CY, Chen A (2012). Predictive factors of in-hospital mortality in colon and rectal surgery. J Am Coll Surg..

[29] Bare M, Monton C, Mora L (2017). COPD is a clear risk factor for increased use of resources and adverse outcomes in patients undergoing intervention for colorectal cancer: a nationwide study in Spain. Int J Chron Obstruct Pulmon Dis..

[30] Rencuzogullari A, Benlice C, Valente M, Abbas MA, Remzi FH, Gorgun E (2017). Predictors of anastomotic leak in elderly patients after colectomy: nomogram-based assessment from the American College of Surgeons National Surgical Quality Program Procedure-Targeted Cohort. Dis Colon Rectum..

[31] Sorokin I, Cardona-Grau DK, Rehfuss A (2016). Stone volume is best predictor of operative time required in retrograde intrarenal surgery for renal calculi: implications for surgical planning and quality improvement. Urolithiasis..

[32] Kim CH, Lee SY, Kim HR, Kim YJ (2017). Nomogram prediction of anastomotic leakage and determination of an effective surgical strategy for reducing anastomotic leakage after laparoscopic rectal cancer surgery. Gastroenterol Res Pract..

[33] Ammendola M, Ruggiero M, Talarico C (2020). No Coil(R) placement in patients undergoing left hemicolectomy and low anterior resection for colorectal cancer. World J Surg Oncol.

[34] Jensen L, Andersen S, Fristrup (1990). Comparison of one dose versus three doses of prophylactic antibiotics, and the influence of blood transfusion, on infectious complications in acute and elective colorectal surgery. Br J Surg.

[35] Bueter M, Thalheimer A, Schuster F (2006). Transfusion-related acute lung injury (TRALI)--an important, severe transfusion-related complication. Langenbecks Arch Surg.

[36] Juliano P, Jean-Louis V, Filomena R (2015). Transfusion requirements in surgical oncology patients a prospective, randomized controlled trial. Anesthesiology..

[37] Weber RS, Jabbour N, Martin RC (2008). Anemia and transfusions in patients undergoing surgery for cancer. Ann Surg Oncol..

[38] Balachandran VP, Gonen M, Smith JJ, DeMatteo RP (2015). Nomograms in oncology: more than meets the eye. Lancet Oncol..

